# Genome sequencing of 15 acid-tolerant yeasts

**DOI:** 10.1128/MRA.00337-23

**Published:** 2023-09-25

**Authors:** James A. Bagley, Michael E. Pyne, Kealan Exley, Kaspar Kevvai, Qingzhao Wang, Malcolm Whiteway, Vincent J. J. Martin

**Affiliations:** 1 Department of Biology, Concordia University, Montréal, Québec, Canada; 2 Centre for Applied Synthetic Biology, Concordia University, Montréal, Québec, Canada; 3 bp Bioscience Center, San Diego, California, USA; University of California, Riverside, California, USA

**Keywords:** yeasts, acid resistance, *Pichia*, *Wickerhamiella*, *Saccharomyces*, *Kazachstania*, *Scheffersomyces*, *Wickerhamomyces*, *Candida*

## Abstract

We report draft genome sequences for 15 non-conventional Saccharomycotina yeast strains obtained from public culture repositories. Included in our collection are eight strains of *Pichia* with broad tolerance to dicarboxylic acids. The genome sequences of these strains will enable comparative genomics of acid-tolerant phenotypes and strain engineering of non-conventional hosts.

## ANNOUNCEMENT

Brewer’s yeast (*Saccharomyces cerevisiae*) is the workhorse of industrial biotechnology owing to its long-standing role in ethanol fermentation and the abundance of sophisticated genetic tools for strain engineering. However, *S. cerevisiae* is not tolerant to high concentrations of many desirable bioproducts or high temperatures, prompting a search for non-conventional hosts with superior tolerance to such conditions. For instance, *Pichia kudriavzevii* is an acid-tolerant yeast (1), *Kluyveromyces marxianus* is a fast-growing thermotolerant host (2), and *Yarrowia lipolytica* is an oleaginous yeast for the production of fatty acids and alkanes (3). Beyond those already characterized in the literature, public culture collections contain thousands of yeast strains that can be mined for specialized phenotypes (4), providing new avenues for host selection beyond the traditional *Saccharomyces*. In a concurrent study, we screened 108 strains of yeast for tolerance to diverse organic acids (5). Here, we report the draft genome for 15 of these strains with tolerance to several dicarboxylic acids. Raw sequence reads are available for all 13 assembled genomes as well as for two strains that could not be assembled into a haploid or phased genome.

A single colony of each strain was inoculated into 10 mL of synthetic complete medium and left to grow at 30°C for 16–20 hours. DNA from fresh cultures was isolated with the QIAGEN Genomic DNA Buffer set in combination with QIAGEN 20/G Genomic tips following the protocol provided by the QIAGEN Genomic DNA Handbook. DNA concentration was measured with the Quant-IT Picogreen dsDNA assay kit and spectrophotometry. DNA purity was assessed via spectrophotometry, and the size and integrity of DNA were assessed using an Agilent TapeStation, no shearing or size selection was applied to the extracted DNA. The extracted DNA was sequenced at the Centre d'expertise et de services Génome Québec using PacBio SMRT technology, and genome sequences were assembled using the PacBio SMRTpipe (HGAP 4 SMRTLink v. 8.0.0). Genome assemblies for most yeast strains yielded less than 30 contigs (Table 1). Two of the strains, *Kazachstania exigua* NCYC 1478 and *Pichia membranifaciens* NCYC 55, resulted in assemblies with approximately double the expected genome size and number of contigs, suggesting that these strains are heterozygous diploids. For these strains, the raw reads are hosted on the NCBI Sequence Read Archive, but the attempted assemblies are not available on GenBank. BUSCO completeness scores were generated using BUSCO 5.4.5 (6) using Saccharomycetes as the taxon, transcriptome mode, and the e-value threshold set to 10E-3. OrthoDB v10 (7) was used as the underlying dataset.

**TABLE 1:** Summary of genome sequencing performed in this study

Species	Depositor ID	Genome size (Mbp)	GC content (%)	Contigs	Max contig size (Mbp)	N50 contig	BUSCO genome assembly completeness scores^a^					Assembly accession	SRA accession
							Complete single-copy	Complete duplicated		Fragmented	Missing		
*Pichia occidentalis*	Y-7552	12.6	40	27	4.3	2.2 Mb	2005	18		52	62	GCA_029581985.1	SRX20007293
*Pichia occidentalis*	Y-6545	11.9	40	12	2.8	1.6 Mb	2031	6		48	52	GCA_029581965.1	SRX20007300
*Pichia occidentalis*	YB-3389	11.7	40	12	2.3	1.3 Mb	2014	12		45	66	GCA_029581915.1	SRX20007299
*Pichia kudriavzevii*	Y-7551	11	38	10	2.7	2 Mb	1966	8		79	84	GCA_029581905.1	SRX20007307
*Pichia fermentans*	Y-1879	13.7	41.5	62	1.7	656 kb	1776	68		134	159	GCA_029581925.1	SRX20007305
*Pichia kluyveri*	05–608 Phaff	11.3	28.5	20	3	2.4 Mb	1902	5		89	141	GCA_029581865.1	SRX20007306
*Pichia membranifaciens*	55 NCYC	19.4	-	-	-	-	-	-		-	-	-	SRX20007303
*Pichia manshurica*	Y-27978	12	42	6	3.4	2.8 Mb	2059	5		28	45	GCA_030220005.1	SRX20007295
*Wickerhamiella sorbophila*	Y-7921	8.3	48.5	10	2.5	1.1 Mb	1910	17		68	142	GCA_029581845.1	SRX20007302
*Saccharomyces cerevisiae*	3040 NCYC	12	38	18	1.5	809.2 kb	2024	47		44	22	GCA_029581825.1	SRX20007296
*Saccharomyces cerevisiae*	Y-144	11.8	38	18	1.5	798.5 kb	2062	53		14	8	GCA_029581805.1	SRX20007301
*Kazachstania exigua*	1478 NCYC	26	-	-	-	-	-	-		-	-	-	SRX20007304
*Scheffersomyces amazonensis*	12363 CBS	13.9	31.5	27	3.4	1.9 Mb	1957	2		3	175	GCA_029581795.1	SRX20007297
*Wickerhamomyces anomalus*	Y-366	15.1	34.5	30	1.8	1.1 Mb	1703	141		193	100	GCA_029581785.1	SRX20007298
*Candida boleticola*	7847 CBS	16.1	44.5	25	3	1.8 Mb	2127	2		2	6	GCA_029581775.1	SRX20007294
													

^a^ BUSCO scores are not provided for *Kazachstania exigua* NCYC 1478 and *Pichia membranifaciens* NCYC 55 since these strains yielded assemblies with approximately double the expected genome size and number of contigs.

## Data Availability

This Whole Genome Shotgun project has been deposited at GenBank under the BioProject accession PRJNA870168. Raw reads for all 15 strains and assembled genomes of 13 strains can be accessed under the BioSample accessions SAMN30353783-SAMN30353798.
